# VHH-based antigen recognition programs Claudin18.2 CAR-T cells for durable activity in low-antigen solid tumors

**DOI:** 10.1186/s12967-026-08611-2

**Published:** 2026-07-22

**Authors:** Dan He, Xiaoyuan Hu, Jiyin Guo, Jiaxing Cheng, Weike Li, Sidi Wan, Yunshan Ye, Lei Zhu, Li Li, Ke Li, Chunlei Ge, Tao Li, Hong Yao

**Affiliations:** Cancer Biotherapy Center & Cancer Research Institute, The Third Affiliated Hospital of Kunming Medical University, Yunnan Cancer Hospital, Peking University Cancer Hospital Yunnan, Kunming, 650106 China

**Keywords:** Gastric cancer, Claudin18.2, CAR-T, Nanobody, VHH, scFv

## Abstract

**Background:**

Claudin18.2 is a clinically validated target for gastric and gastroesophageal junction adenocarcinoma. However, the antigen heterogeneity, low surface density, and issues related to scFv-associated conformational instability and tonic signaling may limit the durability and therapeutic window of Claudin18.2-directed CAR-T cell therapy. Therefore, improving the design of CAR-T cells targeting Claudin18.2 could enhance their effectiveness in treating these cancers.

**Methods:**

We generated third-generation Claudin18.2 CAR-T cells using either a humanized camelid single-domain antibody (VHH) or a conventional scFv as the antigen-recognition module, maintaining an identical CAR backbone for both constructs. To evaluate their performance, we used a gastric cancer cell-line panel with graded Claudin18.2 expression and corresponding xenograft models. We assessed CAR-T cell activity by measuring antigen-dependent cytotoxicity, cytokine secretion (IFN-γ and TNF-α), and reactivity toward antigen-negative targets. In vivo, we examined tumor control, T-cell infiltration, and CD8⁺ T-cell enrichment, and assessed toxicity. Additionally, we investigated the stability of CAR expression, T-cell expansion, and exhaustion-associated phenotypes. Structural modeling and molecular dynamics simulations were performed to evaluate the stability of the binding interface of the VHH–Claudin18.2 complex relative to the scFv–Claudin18.2 complex.

**Results:**

VHH-based CAR-T cells exhibited superior antigen-dependent activity compared to scFv-based CAR-T cells. VHH-CAR-T cells maintained higher cytotoxicity and IFN-γ and TNF-α secretion even under low antigen density and displayed minimal reactivity against antigen-negative targets. In vivo, VHH-CAR-T cells achieved faster and more durable tumor control, with enhanced intratumoral T-cell infiltration and CD8⁺ T-cell enrichment, without any overt toxicity. Mechanistically, the use of a VHH recognition domain was associated with better preserved surface CAR expression after antigen engagement, expanded more robustly, displayed fewer exhaustion-associated phenotypes, had a higher proportion of central memory T cells, and showed transcriptional enrichment of proliferative and effector programs with restrained exhaustion signatures. Structural modeling and molecular dynamics simulations further confirmed that the VHH–Claudin18.2 binding interface was more stable than that of scFv–Claudin18.2.

**Conclusion:**

Replacing scFv with VHH is associated with enhanced functional performance, persistence, and therapeutic efficacy of Claudin18.2-directed CAR-T. These findings support the further development and translation of VHH-based CAR-T strategies for solid tumor treatment.

**Supplementary Information:**

The online version contains supplementary material available at https://doi.org/10.1186/s12967-026-08611-2.

## Introduction

Gastric cancer remains one of the most prevalent malignancies worldwide and continues to be a leading cause of cancer-related mortality despite advances in diagnosis and treatment. According to global cancer statistics, gastric cancer ranks fifth in incidence and fourth in cancer-related deaths, reflecting its substantial disease burden [1]. Gastric carcinogenesis is a multifactorial process associated with environmental exposures, dietary habits, Helicobacter pylori infection, genetic susceptibility, and metabolic dysregulation [2]. Owing to its insidious early manifestations, approximately 60% of Chinese patients are diagnosed at the stage of locally advanced disease or distant metastasis, resulting in limited therapeutic options and extremely poor outcomes [3]. In China, the overall 5-year survival rate for gastric cancer is approximately 33%, while survival for advanced-stage disease remains below 20%, underscoring an urgent need for more effective treatment strategies [4, 5].

Claudin18.2, a tight junction-associated transmembrane protein, exhibits highly restricted expression in normal gastric mucosa, where it is sequestered within epithelial tight junctions. During malignant transformation, disruption of epithelial polarity leads to aberrant surface exposure of Claudin18.2, rendering it accessible to therapeutic targeting [6]. Accumulating evidence indicates that Claudin18.2 is expressed in a substantial subset of gastric and gastroesophageal junction adenocarcinomas, with approximately 30–40% of patients exhibiting moderate to high levels of expression [7, 8]. On this basis, multiple Claudin18.2-targeted therapeutic modalities have entered clinical development. Antibody–drug conjugates targeting Claudin18.2 have demonstrated encouraging antitumor activity with manageable toxicity profiles in early-phase clinical trials [9]. More notably, Claudin18.2-directed CAR-T cell therapy has shown promising efficacy in patients with advanced gastric cancer, as exemplified by the phase II CT041-ST-01 study, which reported improved progression-free survival and an acceptable safety profile. Together, these clinical advances establish Claudin18.2 as a compelling target for cellular immunotherapy in gastric cancer [10].

Despite the success of CAR-T cell therapy in hematologic malignancies, its application in solid tumors remains constrained by multiple biological and technical challenges [11]. These include a highly immunosuppressive tumor microenvironment, heterogeneous and often low-density target antigen expression, and inefficient trafficking and infiltration of CAR-T cells into tumor tissues [12–15]. In addition, most currently approved CAR constructs employ single-chain variable fragments (scFvs) as antigen-recognition domains. Although scFvs enable antigen specificity, their artificial linkage of VH and VL domains renders them susceptible to misfolding, aggregation, and spontaneous self-association, which can drive antigen-independent tonic signaling, premature T-cell exhaustion, and impaired persistence [16–18]. Furthermore, the relatively large size and conformational flexibility of scFv-based CARs may limit effective tumor penetration and contribute to off-tumor or on-target/off-tumor toxicities in solid tumor settings [19–21]. Collectively, these factors substantially restrict the durability and therapeutic window of scFv-based CAR-T therapies for solid tumors.

Camelid-derived single-domain antibodies (VHHs), also known as nanobodies, have emerged as an attractive alternative antigen-recognition platform for CAR engineering. VHHs naturally lack light chains and consist of a single variable domain, conferring several advantageous properties, including small molecular size, high thermal and conformational stability, strong tissue penetration, reduced propensity for aggregation, and ease of humanization [22–24]. Preclinical studies have increasingly demonstrated that VHH-based CAR-T cells may exhibit enhanced antigen sensitivity, improved tissue infiltration, and reduced exhaustion compared with their scFv-based counterparts [25, 26]. These features suggest that VHH-based recognition modules may overcome key structural and functional limitations inherent to conventional scFv-based CAR designs, particularly in solid tumors characterized by antigen heterogeneity and low target density [27].

In the present study, we developed a Claudin18.2-directed VHH-based CAR-T cell product and systematically compared its functional performance with that of an scFv-based CAR-T construct sharing an identical intracellular signaling backbone. Using in vitro models spanning a range of Claudin18.2 expression levels and corresponding in vivo xenograft models, we evaluated antitumor activity, antigen sensitivity, tumor infiltration, and safety profiles. In addition, we investigated phenotypic, transcriptional, and structural features associated with differential CAR-T cell behavior, including exhaustion status, memory differentiation, and receptor–antigen interaction dynamics. Our findings demonstrate that VHH-based Claudin18.2 CAR-T cells exhibit enhanced functional persistence and antitumor efficacy under low-antigen conditions, supporting their potential translational value for the treatment of Claudin18.2-positive gastric cancer.

## Results

### Establishment of a Claudin18.2 expression–gradient model and characterization of CAR expression and antigen recognition

**Fig. 1:**
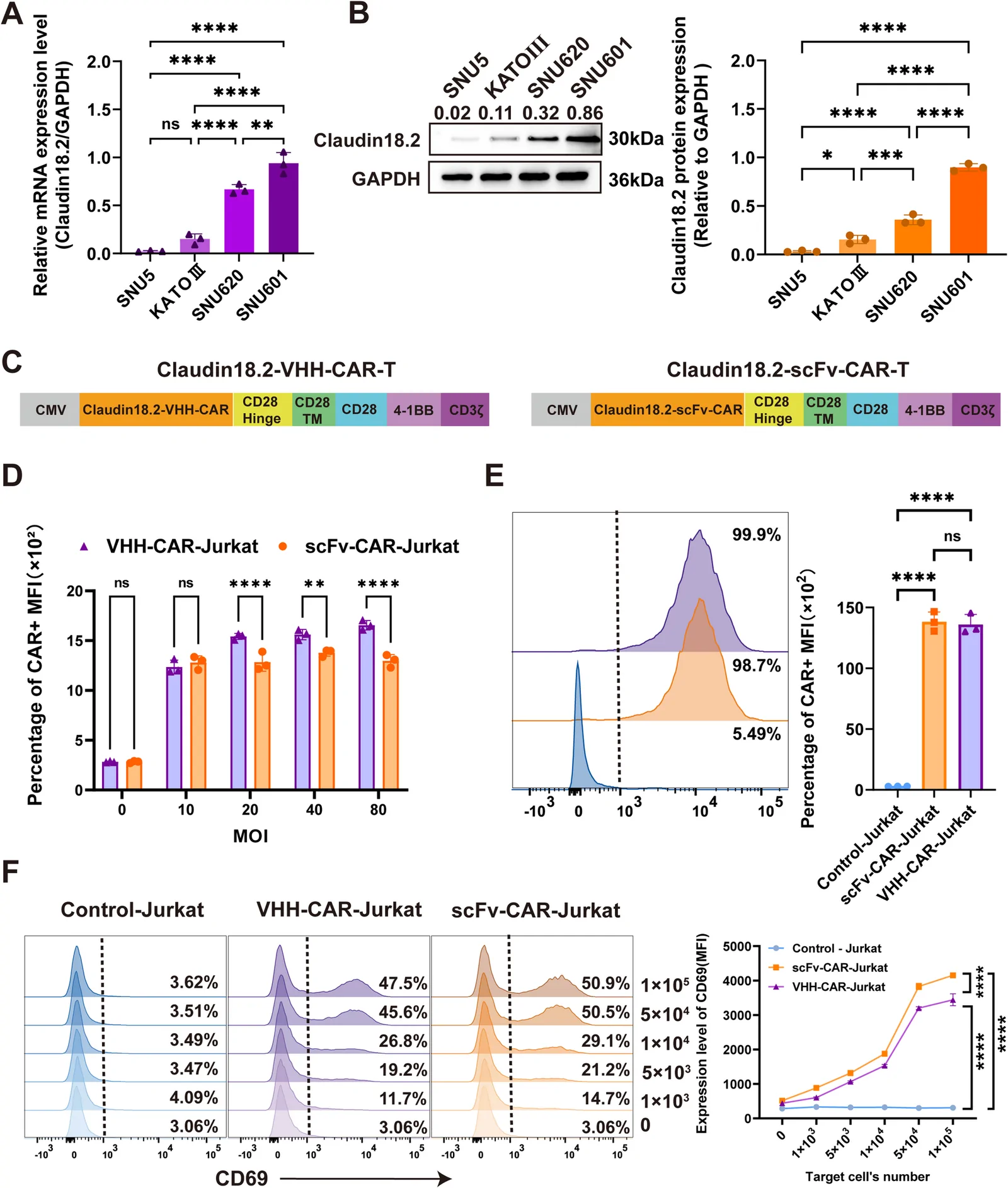
Establishment of a Claudin18.2 expression gradient and evaluation of expression and activation induced by two Claudin18.2-CAR constructs. (**A**) Relative Claudin18.2 mRNA expression in SNU601 (H, high), SNU620 (M, intermediate), KATO-III (L, low), and SNU5 (N, negative) cells as determined by RT–qPCR. Bar graphs represent mean ± SEM. (**B**) Representative Western blot showing Claudin18.2 protein expression in the four cell lines; the right panel shows densitometric quantification of band intensity (Claudin18.2/GAPDH). (**C**) Schematic modular designs of Claudin18.2-targeting scFv-CAR and VHH-CAR constructs (both share the same signal peptide, transmembrane region, and costimulatory/activation domains; only the antigen-binding domain differs). (**D**) Following lentiviral transduction of Jurkat cells, cell-surface CAR expression was assessed by binding to soluble full-length Claudin18.2 protein (directly FITC-labeled); the trends of CAR positivity/expression levels across different MOIs are shown. (**E**) After enrichment of CAR-positive transduced cells using anti-FITC magnetic beads, flow cytometry was used to evaluate CAR surface expression. (**F**) CAR-Jurkat cells at different input numbers (0, 1 × 103, 5 × 103, 1 × 104, 5 × 104, 1 × 105) were co-cultured with SNU601 (**H**) cells for 18 h, after which CD69 expression on Jurkat cells was measured by flow cytometry to assess antigen recognition–induced early activation. Statistical analysis: Mean ± SEM (n = 3 biological replicates). One-way or two-way ANOVA with Tukey’s post hoc test. **P < 0.01, ****P < 0.0001; ns, not significant

To model clinically relevant antigen heterogeneity, we established a gastric cancer cell-line panel spanning a graded range of Claudin18.2 (CLDN18.2) expression. Quantitative RT–PCR and Western blot analyses confirmed a stepwise decrease in Claudin18.2 abundance across SNU601 (high), SNU620 (intermediate), KATO-III (low), and SNU5 (negative) cells (Fig. 1A-B). This panel recapitulates both interpatient and intratumoral heterogeneity of Claudin18.2 expression observed in gastric cancer.

We next generated third-generation Claudin18.2-directed CAR constructs incorporating either a humanized single-domain nanobody (VHH; Hu7v3) [28] or a conventional single-chain variable fragment (scFv; Hu8E5) as the antigen-binding domain, while maintaining identical hinge, transmembrane, and intracellular signaling modules to isolate the effect of the recognition domain (Fig. 1C). When expressed in Jurkat cells, VHH-CAR consistently achieved a higher proportion of CAR-positive cells and greater surface CAR expression across a range of multiplicities of infection (MOIs) compared with scFv-CAR (Fig. 1D). Transduction efficiency reached a plateau at MOI = 20 without compromising cell viability (Fig. 1D; Figure S1); Within the tested MOI range, the VHH-CAR consistently yielded a higher proportion of CAR-positive cells and stronger surface expression overall (Fig. 1D). Given that transduction efficiency approached a plateau at MOI = 20 while cell viability remained stable (Fig. 1D; Supplementary Figure 1); therefore, this condition was used for all subsequent experiments. To further minimize inter-experimental variability, CAR-expressing cells were enriched by anti-FITC magnetic selection, yielding a stable CAR-positive fraction exceeding 90% (Fig. 1E).

Under conditions of matched CAR positivity, antigen-dependent recognition was evaluated using a Jurkat-based CD69 activation assay [29]. Upon stimulation with increasing numbers of Claudin18.2-high SNU601 target cells, both VHH-CAR- and scFv-CAR-transduced Jurkat cells exhibited dose-dependent upregulation of the early activation marker CD69 (Fig. 1F; Supplementary Figure 2-3). These results demonstrate that both CAR designs mediate specific, antigen-dependent activation in response to Claudin18.2-expressing target cells, establishing a comparable baseline for subsequent functional comparisons under varying antigen densities.

### VHH-CAR-T exhibits enhanced cytotoxicity and cytokine secretion in an antigen-density gradient model

**Fig. 2:**
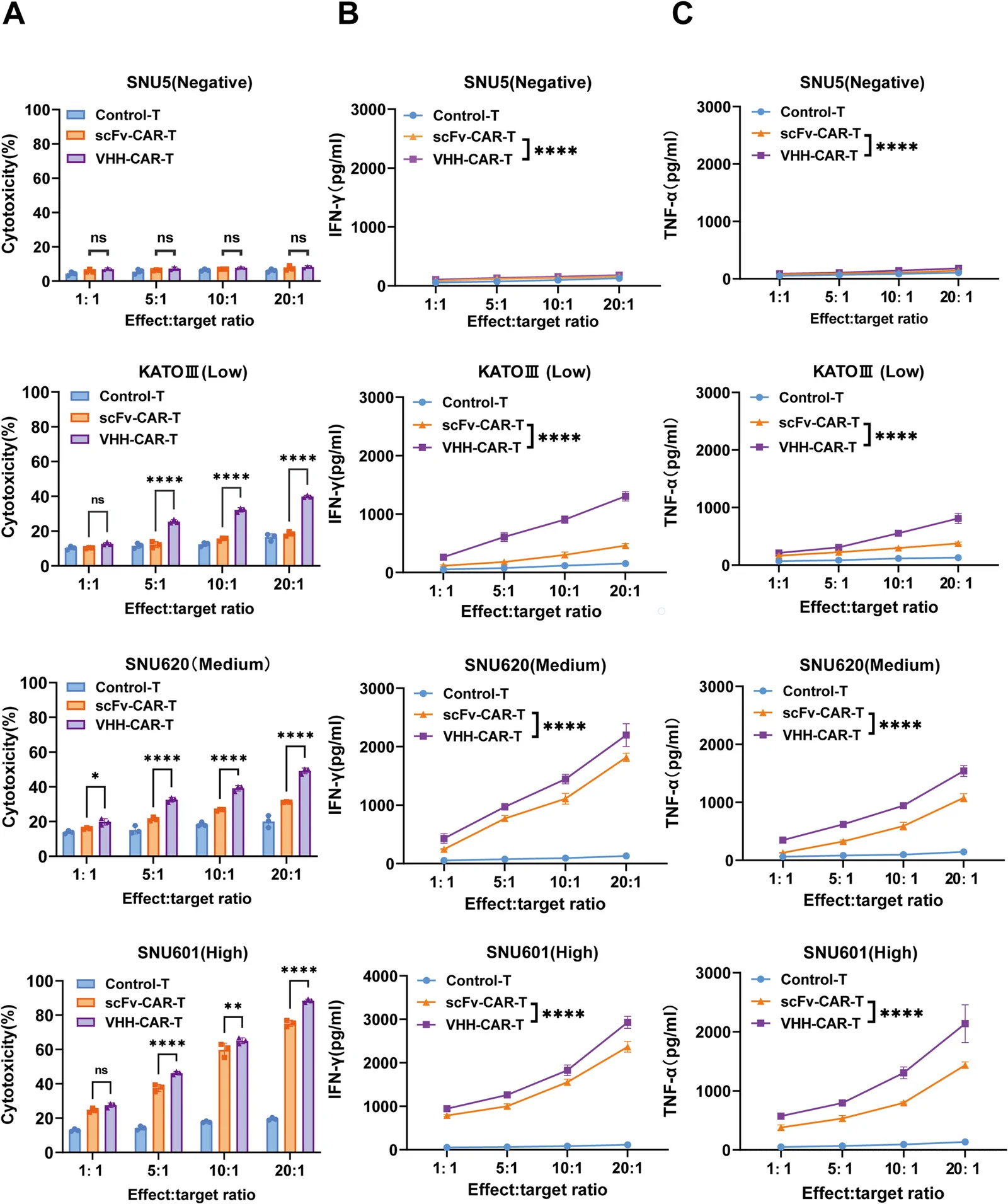
VHH-CAR-T exhibits enhanced in vitro effector function across distinct Claudin18.2 antigen-density conditions. (**A**) Control-T, scFv-CAR-T, and VHH-CAR-T cells were separately co-cultured for 24 h with gastric cancer cell lines exhibiting Claudin18.2 negativity (SNU5), low expression (KATO-III), intermediate expression (SNU620), or high expression (SNU601), and target-cell lysis (cytotoxicity, %) was evaluated at different effector-to-target ratios (E: T = 1:1, 5:1, 10:1, 20:1). (**B**-**C**) Cytokine secretion in co-culture supernatants: IFN-γ (**B**) and TNF-α (**C**) (pg/mL). Statistical analysis: Mean ± SEM (*n* = 3 replicates). Two-way ANOVA with Tukey’s post hoc test. VHH-CAR-T vs. scFv-CAR-T: *P < 0.05, **P < 0.01, ***P < 0.001, ****P < 0.0001; ns, not significant. All functional assays were performed using CAR-enriched T cells with matched surface expression levels (> 90% CAR-positive fraction)

Using the established Claudin18.2 expression–gradient model, we compared the in vitro effector functions of VHH-CAR-T and scFv-CAR-T cells under conditions of high, intermediate, low, and absent antigen expression. Short-term (24 h) cytotoxicity assays were performed against SNU601 (high), SNU620 (intermediate), KATO-III (low), and SNU5 (negative) target cells at multiple effector-to-target (E: T) ratios. Against high- and intermediate-antigen-density targets, both CAR-T products mediated efficient and E: T ratio–dependent tumor cell lysis (Fig. 2A). In contrast, under low-antigen-density conditions, VHH-CAR-T cells consistently exhibited significantly greater cytotoxic activity than scFv-CAR-T cells across multiple E: T ratios (Fig. 2A). Neither CAR-T product induced appreciable cytotoxicity against Claudin18.2-negative SNU5 cells, confirming antigen-dependent specificity of target cell killing.

To further assess effector function, we quantified IFN-γ and TNF-α secretion in co-culture supernatants by ELISA. Upon stimulation with Claudin18.2-positive target cells, VHH-CAR-T cells produced higher levels of both cytokines compared with scFv-CAR-T cells, with the difference most pronounced under low-antigen-density conditions (KATO-III) (Fig. 2B–C). In this setting, scFv-CAR-T cells exhibited minimal cytokine induction, whereas VHH-CAR-T cells mounted a robust polyfunctional response, with significantly elevated IFN-γ and TNF-α secretion (*P* < 0.0001). In contrast, stimulation with Claudin18.2-negative targets failed to elicit cytokine production above background levels in either CAR-T group, further confirming response specificity.

Collectively, these results demonstrate that incorporation of a VHH-based recognition domain enables CAR-T cells to maintain stronger cytotoxic and cytokine responses under conditions of low antigen density, highlighting a functional advantage in antigen-heterogeneous tumor settings.

### VHH-CAR-T exhibits enhanced in vivo antitumor activity in tumor-bearing mouse models

**Fig. 3:**
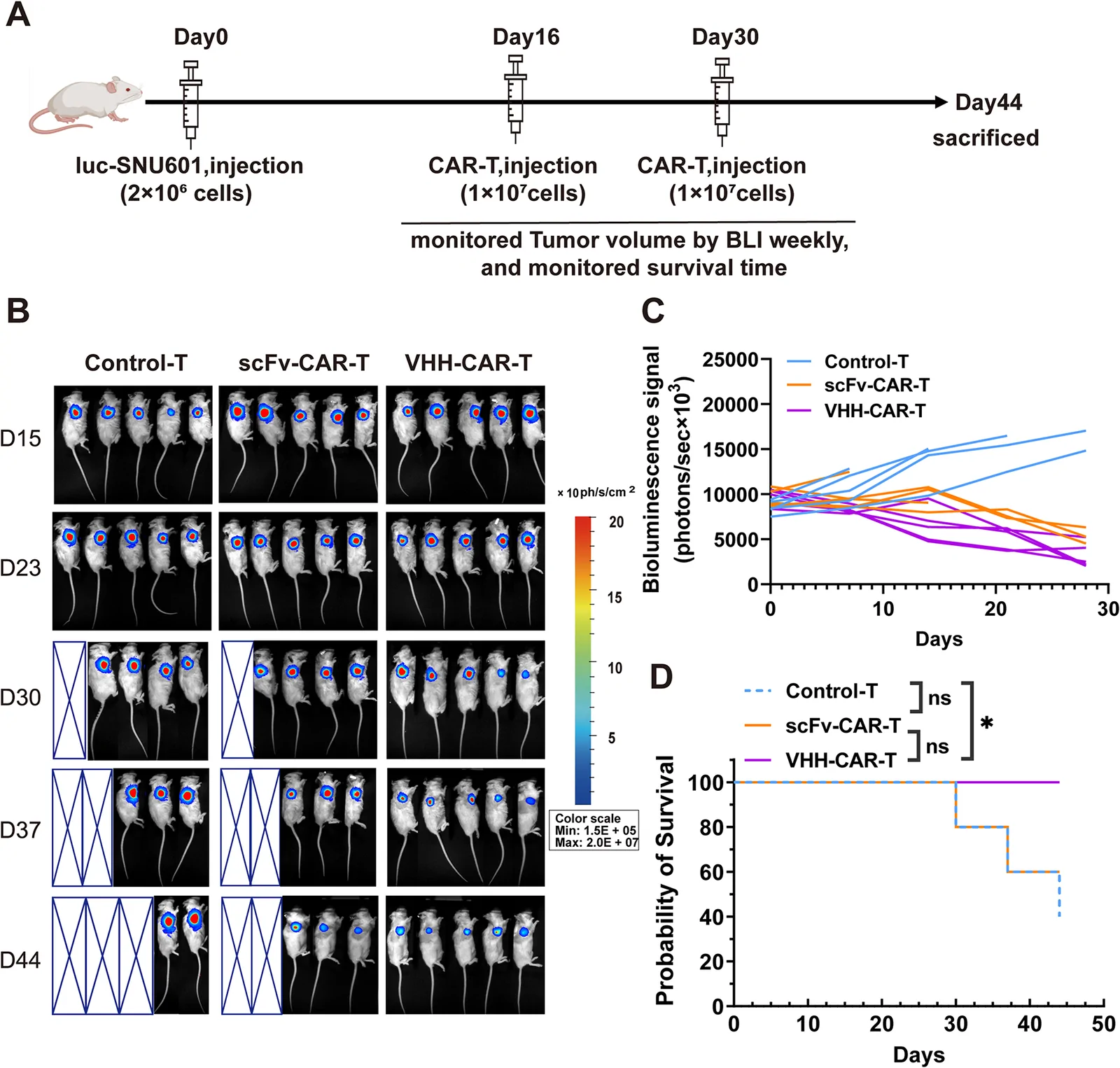
VHH-CAR-T achieves stronger and more durable in vivo antitumor activity in a luc-SNU601 subcutaneous xenograft model. (**A**) Schematic overview of the experimental design and dosing schedule. (**B**) Representative bioluminescence imaging (BLI) images of mice in each group at the indicated time points. Color intensity denotes the tumor-associated bioluminescent signal, with the scale range shown on the right; “×” indicates animals that had died or were removed at endpoint prior to the corresponding time point. (**C**) Longitudinal curves of tumor bioluminescence for individual mice in each group (y-axis, photon flux, photons/sec; each curve represents one mouse). (**D**) Kaplan–Meier survival curves. Statistical analysis: n = 5 mice/group. Survival curves evaluated by log-rank (Mantel-Cox) test (*P = 0.032 for Control-T vs. VHH-CAR-T; ns, not significant)

To assess in vivo antitumor efficacy, a xenograft mouse model was established by subcutaneous implantation of luciferase-expressing SNU601 gastric cancer cells, which exhibit high Claudin18.2 expression. Tumor growth was longitudinally monitored by bioluminescence imaging (BLI). When tumor-associated photon flux reached approximately 8.0 × 10⁴ photons/s, mice were randomized to receive untransduced T cells (Control-T), scFv-CAR-T cells, or VHH-CAR-T cells via tail-vein injection, using CAR-T products with matched and high CAR positivity (≥ 90%) (Fig. 3A).

Longitudinal BLI demonstrated rapid and progressive tumor growth in the Control-T group, whereas both CAR-T–treated groups showed marked suppression of tumor progression (Fig. 3B). Notably, mice treated with VHH-CAR-T cells exhibited a faster and more sustained reduction in tumor-associated bioluminescent signals compared with those treated with scFv-CAR-T cells. Quantitative analysis of photon flux trajectories over time further confirmed that overall tumor burden in the VHH-CAR-T group was significantly lower than that observed in both the Control-T group and the scFv-CAR-T group at the experimental endpoint (Fig. 3C). Although scFv-CAR-T treatment also resulted in significant tumor inhibition relative to controls, residual tumor burden remained consistently higher than that observed in the VHH-CAR-T cohort. Together, these findings indicate that incorporation of a VHH-based recognition domain confers more effective and durable tumor control in vivo.

Survival analysis revealed that mortality events began to occur at approximately day 30 in both the Control-T and scFv-CAR-T groups (Fig. 3D). Mice receiving VHH-CAR-T cells showed a trend toward prolonged survival compared with those receiving scFv-CAR-T cells; however, this difference did not reach statistical significance. Taken together with the tumor growth data, these results suggest that VHH-CAR-T treatment provides improved tumor control with a favorable impact on survival trends in this xenograft model.

Assessment of treatment-related toxicity demonstrated no significant differences in body weight among the three groups throughout the study period. Histopathological examination of major organs, including the heart, liver, spleen, lung, and kidney, revealed no evidence of inflammatory infiltration, tissue necrosis, or structural abnormalities in CAR-T–treated mice (Supplementary Figure 4A–B). These findings indicate that VHH-CAR-T therapy achieves enhanced in vivo antitumor efficacy without detectable systemic toxicity at the administered dose in this preclinical setting.

### VHH-CAR-T cells display enhanced intratumoral infiltration and CD8⁺ T-cell enrichment

**Fig. 4:**
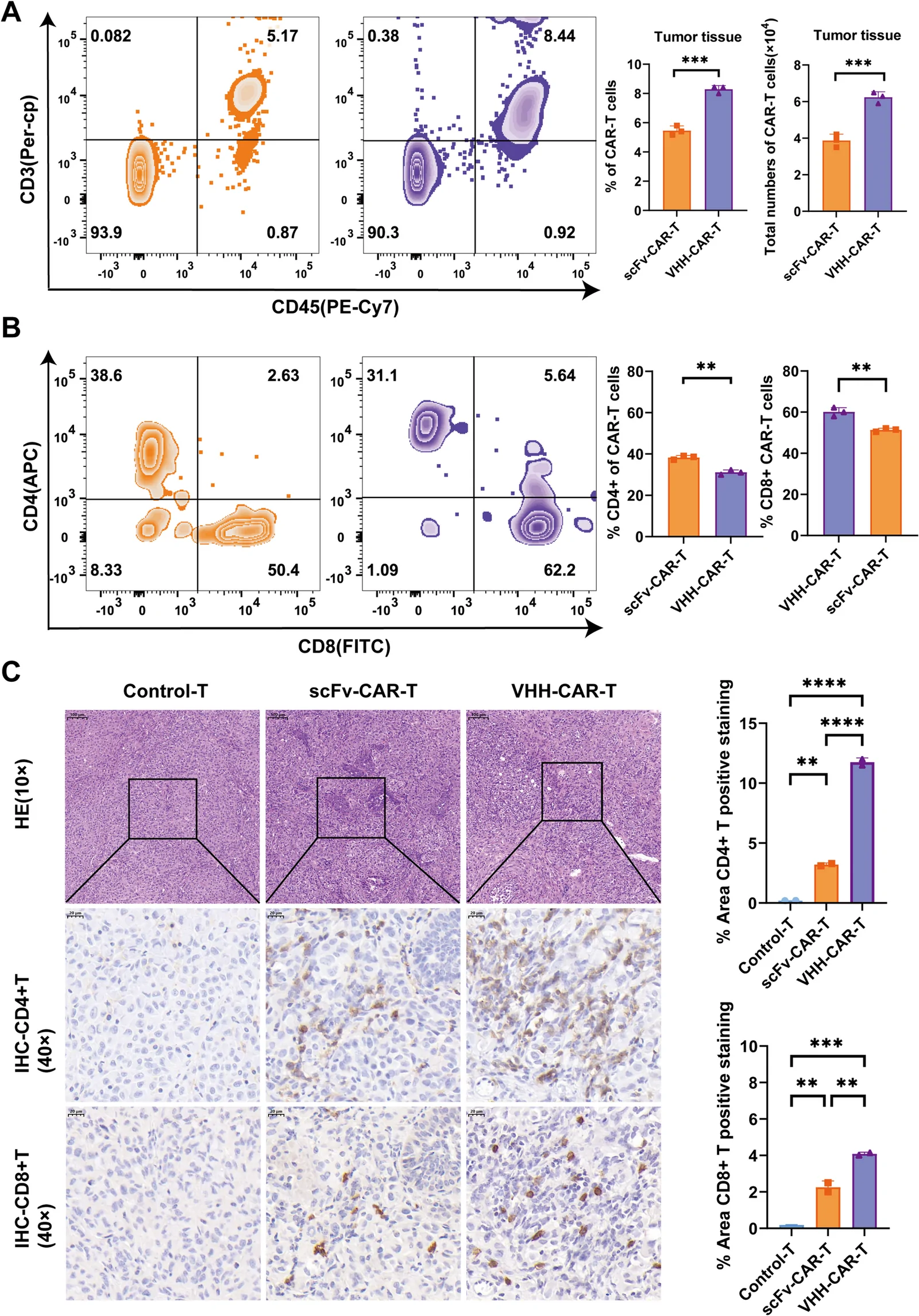
VHH-CAR-T shows enhanced intratumoral infiltration and enrichment of the CD8⁺ lineage. (**A**) Flow-cytometric gating strategy and quantification of CAR-T infiltration in tumor tissues. Left, representative dot plots (CD45-PE-Cy7 × CD3-PerCP) showing the tumor-infiltrating T-cell population; right, quantification of the proportion of CAR-T cells in tumor tissues (% of CAR-T cells) and total CAR-T cell numbers (×10^4^). (**B**) Flow-cytometric analysis of tumor-infiltrating CAR-T cells. Left, representative dot plots (CD8-FITC × CD4-APC); right, quantification of the frequencies of CD4⁺ and CD8⁺ cells within the CAR-T population. (**C**) Tumor histology and IHC validation. Top row, H&E staining (10×) showing tumor architecture; middle and bottom rows, representative fields of CD4 and CD8 IHC staining, respectively (40×). Right, quantification of IHC-positive signals. Data are presented as mean ± SEM (each dot represents one mouse). Statistical analysis: Mean ± SEM. One-way ANOVA with Tukey’s post hoc test. **P < 0.01, ***P < 0.001, ****P < 0.0001

To investigate cellular correlates of the enhanced in vivo antitumor activity observed with VHH-CAR-T treatment, we analyzed intratumoral CAR-T cell infiltration and immunophenotypic composition using flow cytometry and histological approaches. Flow-cytometric analysis of tumor-infiltrating lymphocytes isolated at the study endpoint demonstrated that both the frequency and absolute number of CAR-T cells were significantly higher in tumors from the VHH-CAR-T group than in those from the scFv-CAR-T group (Fig. 4A), indicating improved tumor infiltration and/or retention.

Phenotypic profiling of infiltrating CAR-T cells revealed a marked enrichment of CD8⁺ T cells within the VHH-CAR-T population, accompanied by a relative reduction in CD4⁺ T cells, compared with the scFv-CAR-T group (Fig. 4B). As a result, the intratumoral CD8⁺/CD4⁺ ratio was significantly increased in tumors treated with VHH-CAR-T cells, consistent with a cytotoxic effector–dominant infiltrate.

Histopathological analyses supported these flow-cytometric findings. Hematoxylin and eosin staining, together with immunohistochemical staining for CD4 and CD8, demonstrated more extensive T-cell infiltration within tumor parenchyma in the VHH-CAR-T–treated group compared with both the scFv-CAR-T and Control-T groups (Fig. 4C). In particular, CD8⁺ T cells were more broadly distributed throughout tumor tissues following VHH-CAR-T treatment, corroborating the increased abundance and effector-skewed composition observed by flow cytometry.

Collectively, these data indicate that VHH-CAR-T therapy is associated with enhanced intratumoral accumulation of CAR-T cells and a higher proportion of CD8⁺ effector T cells within the tumor microenvironment, providing a cellular basis for its superior antitumor activity in vivo.

### VHH-CAR-T cells display improved surface CAR stability, expansion, and reduced exhaustion under chronic antigen stimulation

**Fig. 5:**
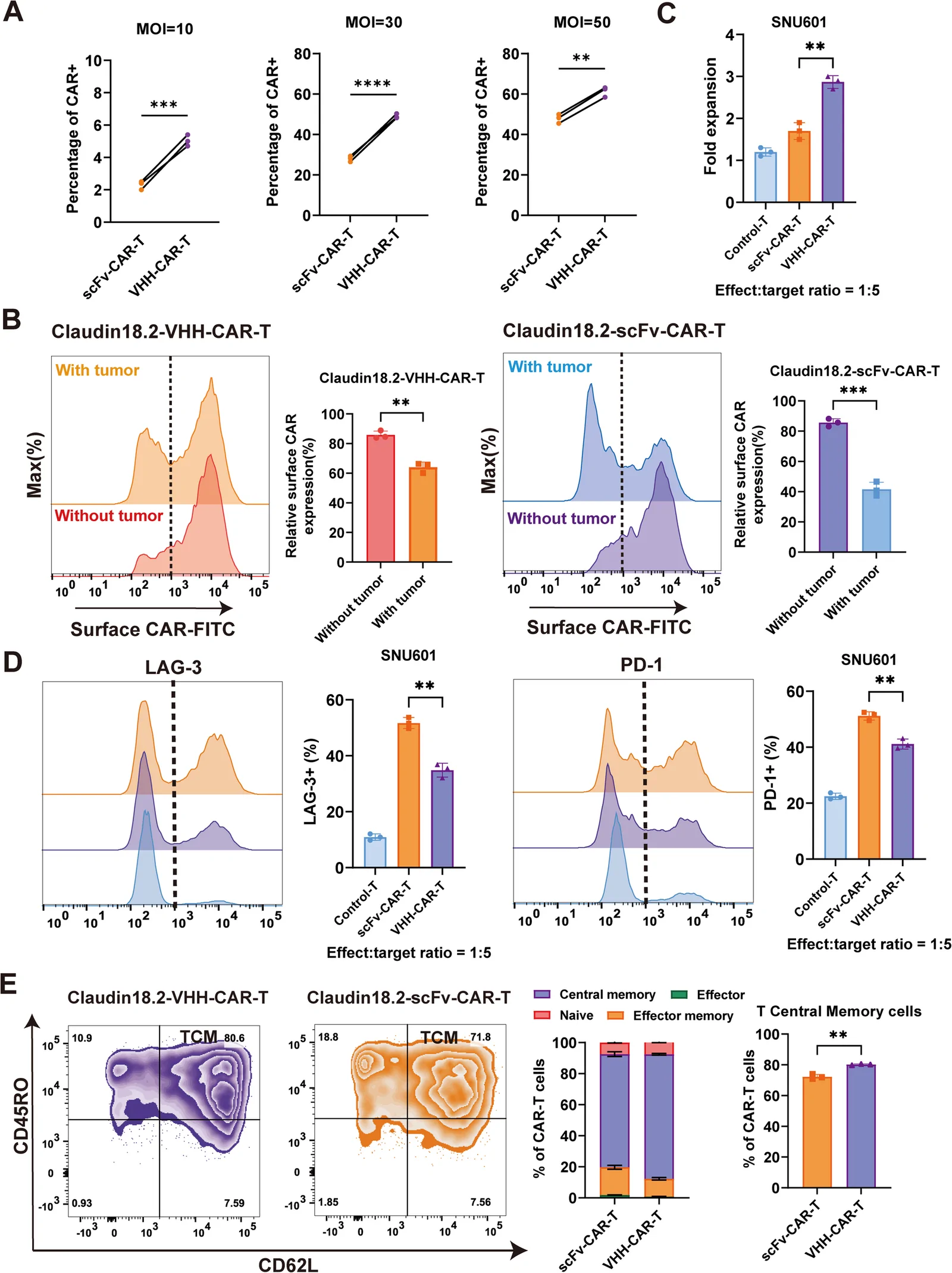
The VHH recognition domain improves CAR transduction efficiency, preserves surface expression after antigen stimulation, and maintains functional homeostasis. (**A**) Claudin18.2-scFv-CAR-T and Claudin18.2-VHH-CAR-T were generated at different lentiviral multiplicities of infection (MOI = 10, 30, 50); transduction efficiency was assessed by flow cytometric quantification of the percentage of CAR⁺ cells. Each dot represents an independent manufacturing run/biological replicate, and lines connect matched controls from the same batch. (**B**) Each CAR-T product was incubated with or without Claudin18.2-positive SNU601 cells for 48 h, followed by measurement of the cell-surface CAR-FITC signal. The left and right panels show representative overlaid histograms for VHH-CAR-T and scFv-CAR-T, respectively; the adjacent bar graph presents the quantitative comparison of relative surface CAR expression (%) (with tumor vs. without tumor). (**C**) After 48 h of co-culture with SNU601 cells at E: T = 1:5, the fold expansion of Control-T, scFv-CAR-T, and VHH-CAR-T was compared. (**D**) Exhaustion-associated phenotypes following sustained antigen stimulation were evaluated by measuring LAG-3 and PD-1 expression; representative overlaid flow plots are shown on the left, and the right panel quantifies the percentage (%) of LAG-3⁺/PD-1⁺ cells (analyzed separately under E: T = 1:5 conditions). (**E**) Differentiation phenotypes after antigen stimulation were assessed by CD62L × CD45RO profiling of CAR-T cells, with representative gating shown (TCM region indicated); the right panel shows stacked bar plots of subset composition (effector/effector memory/central memory/naïve), together with a separate quantification of the proportion of central memory T cells (TCM). Statistical analysis: Mean ± SEM (n = 3 biological replicates). Matched samples analyzed by paired Student’s t-test; multi-group comparisons by one-way ANOVA with Tukey’s post hoc test. **P < 0.01, ***P < 0.001, ****P < 0.0001; ns, not significant.All functional assays were performed using CAR-enriched T cells with matched surface expression levels (> 90% CAR-positive fraction)

To delineate the structural determinants associated with the improved performance of VHH-CAR-T cells, we first evaluated its impact on receptor expression and manufacturing efficiency. Across a range of multiplicities of infection (MOI = 10, 30, and 50), VHH-CAR constructs consistently yielded a significantly higher proportion of CAR+ cells compared to their scFv counterparts. This finding suggests that the single-domain VHH extracellular architecture may facilitate more efficient lentiviral packaging or support more robust protein folding and stable surface translocation (Fig. 5A).

Given that antigen-induced CAR internalization and downregulation represent a major limitation to sustained CAR-T activity in solid tumors [30], we next assessed surface CAR expression following prolonged antigen exposure. After 48 h of co-culture with Claudin18.2-positive SNU601 target cells, both CAR-T products exhibited antigen-dependent reductions in surface CAR levels. However, VHH-CAR-T cells retained significantly higher relative surface CAR expression compared with scFv-CAR-T cells (Fig. 5B), indicating improved maintenance of receptor availability following antigen engagement.

We then examined functional consequences associated with differential CAR surface stability. Under conditions of sustained antigen stimulation (E: T = 1:5), VHH-CAR-T cells underwent significantly greater cellular expansion than scFv-CAR-T cells over the same culture period (Fig. 5C). Phenotypic analysis further revealed that, following chronic stimulation, VHH-CAR-T cells expressed lower levels of the inhibitory receptors PD-1 and LAG-3 relative to scFv-CAR-T cells (Fig. 5D). In parallel, memory differentiation profiling demonstrated that VHH-CAR-T cells maintained a higher proportion of central memory T cells (Tcm) after antigen exposure (Fig. 5E).

Together, these findings indicate that incorporation of a VHH-based recognition domain is associated with improved surface CAR stability, enhanced proliferative capacity, and attenuation of exhaustion-associated phenotypes under chronic antigen stimulation.

### VHH-based recognition is associated with a shift toward a self-renewing effector-memory transcriptional program

**Fig. 6:**
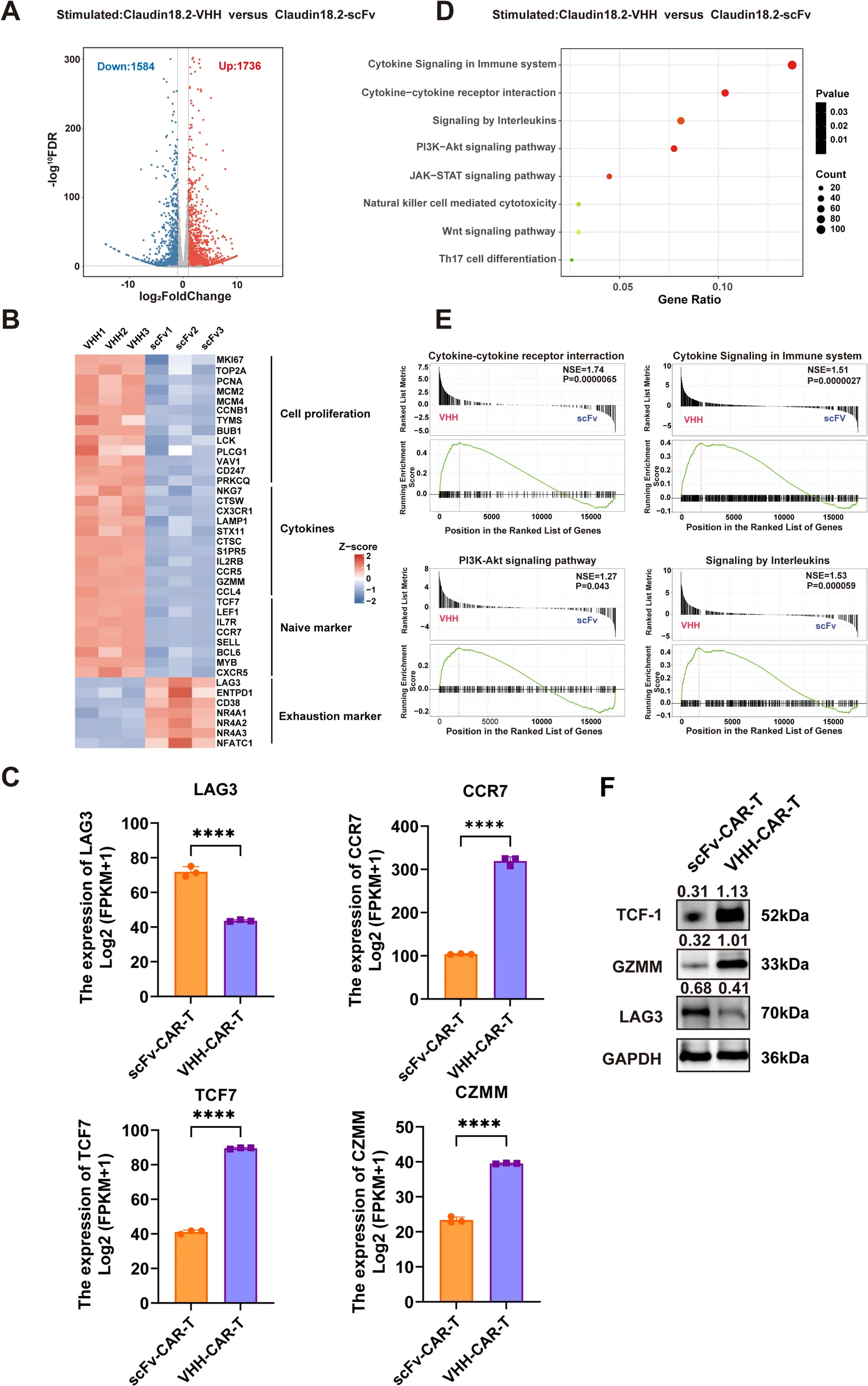
Enhanced effector program and attenuated exhaustion profile in Claudin18.2-VHH-CAR-T cells following antigen stimulation. (**A**) Volcano plot of DEGs (VHH vs. scFv) from RNA-seq (*n* = 3 biological replicates/group) after 48-h co-culture with Claudin18.2-positive target cells (E: T = 1:5). Red: upregulated (1,736 genes); blue: downregulated (1,584 genes). (**B**) Heatmap (Z-score normalized) of representative genes related to proliferation, cytokines, naïve/memory markers, and exhaustion. (**C**) FPKM expression levels of *LAG3*, *TCF7*, *CCR7*, and *GZMM* (mean ± SEM, ****P < 0.0001). (**D**, **E**) Bubble plot (**D**) and GSEA plots (**E**) showing pathway enrichment of DEGs, highlighting cytokine, PI3K-Akt, and interleukin signaling pathways. (**F**) Western blot analysis of TCF-1, GZMM, and LAG-3 protein levels after antigen stimulation, with GAPDH as the loading control. Densitometric values normalized to GAPDH are indicated above the bands. DEGs were identified via edgeR (FDR < 0.05). Pathway/GSEA P-values were adjusted using the Benjamini-Hochberg method (FDR < 0.05)

To dissect the molecular basis of the superior persistence and effector function of VHH-CAR-T cells, we performed global transcriptomic profiling by RNA sequencing (RNA-seq) following chronic antigen stimulation (E: T = 1:5, 48 h). Differential expression analysis identified 1,736 upregulated and 1,584 downregulated genes in VHH-CAR-T cells compared with scFv-CAR-T cells, revealing profound transcriptional divergence (Fig. 6A).

Hierarchical clustering and module-based analysis highlighted that VHH-CAR-T cells exhibited strong induction of cell-cycle regulators (e.g., MKI67, TOP2A, PCNA) and cytotoxic effector molecules (e.g., GZMM, CCL4, NKG7), indicating heightened proliferative and cytolytic readiness (Fig. 6B). Importantly, VHH-CAR-T cells displayed marked enrichment of “stem-like” and memory-associated transcriptional programs, with elevated expression of canonical memory markers and transcription factors, including TCF7 (encoding TCF-1), CCR7, LEF1, and SELL, which are critical for T-cell self-renewal and long-term persistence (Fig. 6B-C). This stem-like transcriptional profile was coupled with significant attenuation of terminal exhaustion signatures, as reflected by reduced expression of inhibitory receptors (LAG3) and key transcriptional drivers of dysfunction (e.g., NR4A family, ENTPD1, CD38) (Fig. 6B-C).

To further probe the upstream signaling networks driving these shifts, we performed Gene Set Enrichment Analysis (GSEA) and pathway enrichment. VHH-CAR-T cells showed robust activation of core immune-potency axes, including cytokine-cytokine receptor interactions, interleukin signaling, and PI3K-Akt and JAK-STAT pathways (Fig. 6D-E). Quantitative GSEA confirmed significant positive enrichment for ‘Cytokine Signaling in the Immune System’ and ‘Signaling by Interleukins’, providing a molecular explanation for the enhanced cytokine secretion and proliferation observed in functional assays (Fig. 6E).

Consistent with these transcriptomic signatures, Western blot analysis showed increased protein expression levels of TCF-1 and GZMM and reduced protein expression levels of LAG-3 in VHH-CAR-T cells compared with scFv-CAR-T cells (Fig. 6F). Flow-cytometric analysis further confirmed a higher frequency of Ki-67⁺ cells in the VHH-CAR-T group after antigen stimulation (Supplementary Figure 5A-B). To functionally validate the RNA-seq-inferred stem-like self-renewal capacity, we performed three consecutive rounds of in vitro antigen rechallenge assays. Although scFv-CAR-T cells rapidly lost cytolytic activity after repeated antigen exposure, VHH-CAR-T cells maintained robust target-cell killing across serial stimulations (Supplementary Figure 5C). This functional assay directly supports the transcriptional enrichment of a durable stem-like effector program in VHH-CAR-T cells.

Collectively, these transcriptomic, protein-level, and functional validation analyses indicate that under chronic antigen stimulation, VHH-CAR-T cells exhibit a profile consistent with a shift toward a self-renewing effector-memory state, combining heightened proliferative and cytotoxic potential with attenuated exhaustion, distinguishing them from scFv-CAR-T cells.

### Structural modeling and MD simulations reveal a more compact and stable VHH–Claudin18.2 complex

**Fig. 7:**
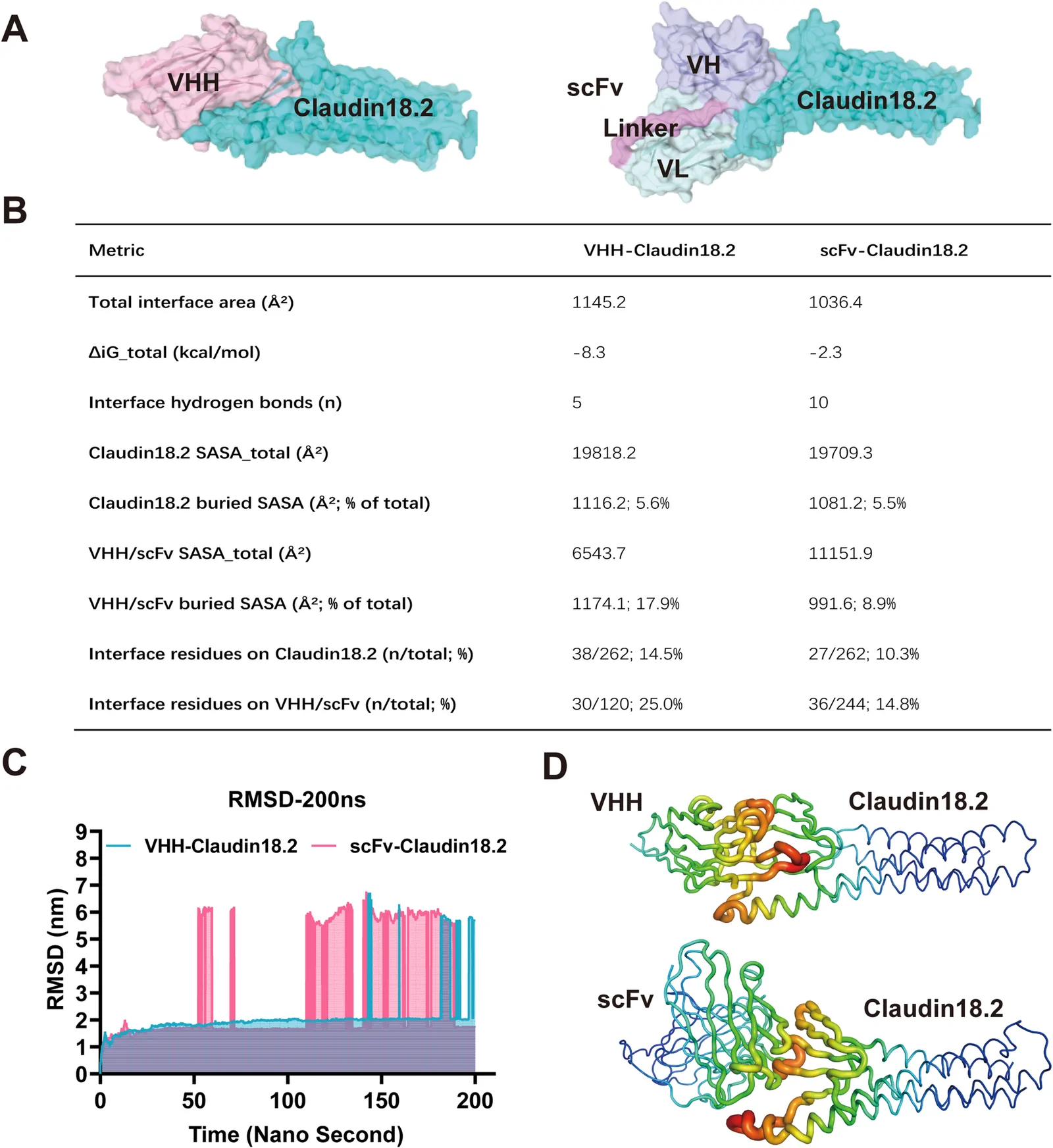
Structural modeling and molecular dynamics analysis of VHH- and scFv-based recognition domains in complex with Claudin18.2. (**A**) Structural models of the VHH-Claudin18.2 and scFv-Claudin18.2 complexes. VHH is a single-domain format, whereas scFv (single-chain variable fragment) comprises VH (variable domain of the heavy chain) and VL (variable domain of the light chain) connected by a peptide linker. (**B**) Interface geometric and energetic parameters calculated using PDBePISA. SASA (solvent-accessible surface area; Å²) denotes the total solvent-accessible surface area, and buried SASA (ΔSASA; Å²) denotes the SASA buried at the interface upon complex formation for each chain. ΔiG_total (kcal/mol) represents the solvation free energy gain upon interface formation (more negative values indicate a more favorable solvation contribution), but it is not equivalent to experimentally measured binding free energy or affinity. (**C**) RMSD (nm) of the VHH-Claudin18.2 and scFv-Claudin18.2 complexes over 200 ns of MD simulation. (**D**) Mapping of B-factor-like displacement metrics derived from the MD trajectories for the VHH-Claudin18.2 and scFv-Claudin18.2 complexes. Structures are rendered as tubes with color and thickness scaled by residue displacement amplitude: thicker/red regions indicate larger displacements (higher flexibility), whereas thinner/blue regions indicate smaller displacements (greater stability). Statistical analysis: Interface parameters (**B**) calculated from a single snapshot via PDBePISA. Trajectories (**C**, **D**) computed over a continuous 200-ns simulation window

To complement functional observations under low antigen density (Fig. 2) and the antigen-stimulation–associated differences in CAR surface expression and phenotypes (Fig. 5), we generated structural models of Claudin18.2 in complex with either the VHH or scFv binding domains and performed 200-ns molecular dynamics (MD) simulations (Fig. 7). In the modeled complexes, VHH adopted a relatively compact binding configuration, concentrating contacts on a localized surface patch of Claudin18.2. By contrast, the scFv-Claudin18.2 complex exhibited a more extended architecture, with interactions distributed across a broader surface region (Fig. 7A), indicating that binding formats can differ in interface organization even when targeting the same antigen.

Interface analysis using PDBePISA revealed a total interface area of 1,145.2 Å² for VHH-Claudin18.2 versus 1,036.4 Å² for scFv-Claudin18.2, with corresponding ΔiG_total values of − 8.3 and − 2.3 kcal/mol, respectively (Fig. 7B). Given the size differences between VHH and scFv, we also compared normalized interface metrics based on solvent-accessible surface area (SASA). On the recognition-domain side, the buried SASA fraction was 17.9% for VHH (1,174.1/6,543.7 Å²) versus 8.9% for scFv (991.6/11,151.9 Å²), and the fraction of interface residues was higher for VHH (30/120, 25.0%) compared with scFv (36/244, 14.8%). On the antigen side, Claudin18.2 exhibited comparable buried SASA fractions in both complexes (5.6% vs. 5.5%), indicating that the primary difference lies in the extent of binding module engagement at the interface (Fig. 7B). Interestingly, the scFv–Claudin18.2 interface contained more predicted hydrogen bonds (10 vs. 5), highlighting differences in interface composition despite the higher normalized interface contribution for VHH.

Conformational flexibility and stability assessed by molecular dynamics (MD) simulations further supported these structural observations. Over a 200-ns simulation window, the Root-Mean-Square Deviation (RMSD) trajectories showed pronounced, early large-amplitude fluctuations in the scFv–Claudin18.2 complex (at ~ 50 ns). Conversely, the VHH–Claudin18.2 complex exhibited smaller-amplitude RMSD variations, indicative of reduced structural flexibility and a more compact, well-accommodated binding interface under simulated aqueous environments (Fig. 7C). B-factor–like displacement analysis revealed distinct local mobility patterns: the VHH–Claudin18.2 complex displayed localized, healthy flexibility within the VHH loop region near the interface while the Claudin18.2 backbone remained stable. In contrast, the scFv–Claudin18.2 complex imposed increased structural displacement onto the Claudin18.2 interface itself against a rigid scFv framework (Fig. 7D).

It is important to clarify that the primary objective of employing 200-ns MD simulations was not to computationally calculate absolute thermodynamic binding free energies or substitute for cell-free biophysical assays. Rather, these simulations were implemented to investigate the spatial accessibility, orientation, and structural dynamics of the single-domain VHH architecture relative to the multi-domain paired scFv structure upon antigen engagement. These dynamic models suggest that the VHH module maintains a more balanced and rigid orientation when complexed with Claudin18.2, offering a potential structural mechanism for the enhanced persistence and lower receptor downregulation observed in our cell-based functional assays.

## Discussion

Despite incremental survival gains brought by targeted agents and immunotherapies in gastric cancer and GEJC, advanced patients who progress after multiple lines of treatment face a severe lack of viable options [31]. Claudin18.2-directed CAR-T cell therapy represents a potent intervention, but its long-term durability in solid tumors is continuously limited by antigen heterogeneity and scFv-associated liabilities such as tonic signaling [32, 33]. To address these structural and functional vulnerabilities, our study provides a direct, head-to-head evaluation where the CAR signaling backbone was held strictly constant, isolating the antigen-recognition domain format (humanized VHH versus conventional scFv) as the sole variable [34, 35]. Our systematic characterization demonstrates that replacing the scFv with a VHH module is associated with optimized CAR expression stability, anti-tumor sensitivity, and cellular persistence. This controlled structural comparison establishes a foundational framework for understanding how single-domain antibody architectures can overcome the intrinsic limitations of conventional scFv designs in solid malignancies.

A major hurdle in solid-tumor CAR-T therapy is target antigen heterogeneity, where low-antigen-density niches frequently fail to trigger sufficient CAR signaling, leading to immune evasion [36]. Additionally, CAR signaling output is closely coupled with surface receptor availability, which can compound functional deficits under limiting antigen exposure [37, 38]. To address these spatial and temporal challenges, we established a Claudin18.2 antigen-density gradient to systematically compare single-domain VHH- versus conventional scFv-based CAR architectures on an identical signaling backbone (Fig. 1). Our data demonstrate that under limiting antigen availability, VHH-CAR-T cells maintain superior, antigen-specific cytotoxicity and polyfunctional cytokine secretion (IFN-γ and TNF-α) without sacrificing specificity. (Fig. 2). This format-dependent advantage supports the broader concept that the compact single-domain VHH architecture lowers the threshold for functional activation under low-density antigen conditions [39]. While our cell-line panel and subcutaneous models cannot fully capture the spatial-temporal antigen heterogeneity of clinical tumors, they offer vital mechanistic insights into format-dependent CAR-T activation thresholds. In heterogeneous in vivo environments where scFv-CAR-T cells often exhibit functional deficits against low-antigen niches, VHH-CAR-T cells demonstrate superior antigen sensitivity, sustained receptor stability, and an enriched effector-memory program. This suggests that the VHH architecture is theoretically better equipped to eliminate low-antigen clones and mitigate antigen escape. Nonetheless, transitioning these findings into clinical strategies will require rigorous evaluation in advanced, heterogeneous platforms like PDX, orthotopic models, and patient-derived organoid co-cultures.

Beyond antigen sensitivity, CAR-T persistence and functional durability are intrinsically linked to the biophysical properties and baseline signaling of the receptor architecture. Conventional scFv-based CARs are frequently prone to antigen-independent clustering and tonic signaling—liabilities that accelerate T-cell differentiation and terminal exhaustion [40, 41]. In contrast, our study reveals that the single-domain VHH architecture correlates with improved CAR expression stability and surface availability following antigen engagement (Fig. 5). Functionally, this superior receptor stability manifests as enhanced cellular expansion under chronic stimulation, coupled with a robust central memory (Tcm) phenotype and a transcriptional program enriched for proliferative and effector pathways while suppressing exhaustion signatures (Figs. 5 and 6). These findings align with emerging engineering strategies that aim to mitigate T-cell exhaustion by optimizing the antigen-binding domain’s biophysical framework.

The physiological relevance of these format-dependent advantages was validated in vivo, where VHH-CAR-T cells mediated more rapid and durable tumor regression in NSG xenograft models compared to their scFv counterparts (Fig. 3). This enhanced antitumor efficacy was underpinned by superior intratumoral accumulation and a higher CD8⁺/CD4⁺ T-cell ratio within the tumor microenvironment (Fig. 4). However, our in vivo evaluation was performed in an immunodeficient subcutaneous xenograft model, which does not fully recapitulate orthotopic gastric tumor architecture or the complexity of the human immune microenvironment. Future studies should therefore validate these findings in orthotopic and metastatic models, patient-derived xenografts, or organoid–immune co-culture systems, and should rigorously assess gastric mucosa–associated on-target/off-tumor risks [42–44]. In addition, no significant systemic toxicity was detected by weight monitoring or histologic analysis of major organs, which supports the feasibility of further preclinical safety evaluation (Supplementary Figure 4). Although this does not address the potential on-target/off-tumor toxicity caused by Claudin18.2 expression in normal gastric tissue, it supports the feasibility of further safety evaluations. Furthermore, we did not perform a comprehensive in vitro cross-reactivity and off-target screening assay for this VHH candidate gene. Future studies using human Claudin18.2 knock-in models or organoid co-cultures, as well as high-throughput membrane proteome arrays, are needed to rigorously validate their safety prior to clinical translation.

To elucidate the molecular basis for the enhanced antigen sensitivity (Fig. 2) and phenotypic stability (Figs. 5 and 6) of VHH-CAR-T cells, we evaluated the receptor-antigen interface using structural modeling and molecular dynamics (MD) simulations (Fig. 7). These analyses revealed that the single-domain VHH architecture forms a highly stable and compact interface with Claudin18.2. Compared to the scFv format, the VHH complex is characterized by a greater normalized buried surface fraction, more favorable solvation energy (ΔiG_total), and markedly reduced conformational fluctuations (Fig. 7). By circumventing the intrinsic inter-domain flexibility typical of scFvs, the rigid VHH module may structurally optimize downstream CAR signaling mechanics, thereby sustaining robust activation under limiting antigen conditions. While these computational insights compellingly link receptor biophysics to our functional observations, they rely on geometric approximations and omit spatial complexities such as membrane anchoring and glycosylation. Beyond these computational limitations, we acknowledge that the mechanisms driving the robust performance of VHH-CAR-T cells in low-antigen environments are inherently multifactorial. Specifically, differences in the intrinsic affinity of the Hu7v3 VHH domain compared to the Hu8E5 scFv, as well as distinct epitope accessibility on the Claudin18.2 molecule, may also contribute to the observed superiority in CAR-T cell activation and persistence. Furthermore, although our experiments rigorously normalized baseline CAR expression via magnetic sorting, the distinct biophysical properties of the VHH structure itself could differentially modulate receptor stability and internalization kinetics. Therefore, explicitly establishing causality will necessitate not only membrane-aware simulations, but also future empirical studies employing affinity-tuned variants and domain-swapped constructs to delineate the precise contribution of each parameter to the enhanced therapeutic efficacy observed here.

**Fig. 8:**
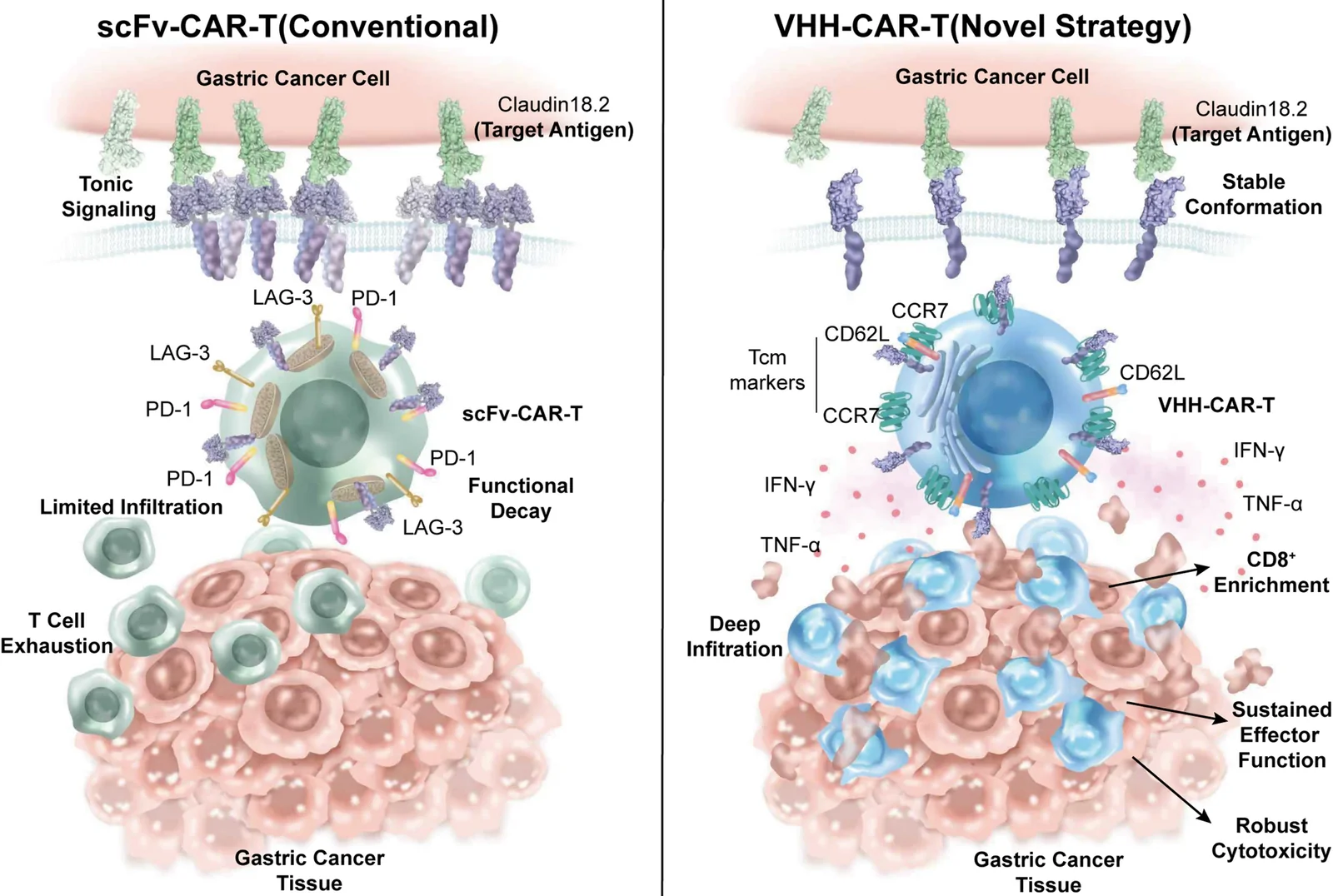
Comparison of scFv-CAR-T vs. VHH-CAR-T in Gastric Cancer Treatment. The left panel illustrates the scFv-CAR-T approach, showing limited tumor infiltration and functional decay, as indicated by T-cell exhaustion markers (PD-1, LAG-3). The right panel depicts the VHH-CAR-T strategy, highlighting deeper tumor infiltration, increased CD8 + T-cell enrichment, and sustained effector function, including higher cytokine secretion (IFN-γ, TNF-α) and memory T-cell markers (CCR7, CD62L)

In summary, this head-to-head evaluation establishes the humanized single-domain VHH architecture as a superior antigen-recognition module that optimizes the functional durability of Claudin18.2-directed CAR-T cell therapy in gastric cancer. Mechanistically, as illustrated in our conceptual model (Fig. 8), the compact VHH interface may help circumvent the intrinsic scFv liabilities of tonic signaling and rapid receptor downregulation, potentially contributing to a shift in the T-cell fate away from terminal exhaustion toward a self-renewing central memory phenotype with robust polyfunctional cytokine secretion. Given that target antigen heterogeneity remains a primary barrier to successful solid tumor immunotherapy, this structural refinement offers a highly translatable strategy to prevent immune evasion and achieve sustained antitumor efficacy [45, 46].

## Materials and methods

### Cell lines

HEK293T cells were obtained from the American Type Culture Collection (ATCC; CRL-3216) and maintained in DMEM (#C11995500CP, Gibco) supplemented with 10% fetal bovine serum (FBS; #10099–141 C, Gibco) and 1% penicillin-streptomycin (SV30010, HyClone). Jurkat and SNU601 cells were purchased from Procell (Pricella, Wuhan, China) and cultured in RPMI-1640 (#11875-093, Gibco) supplemented with 10% FBS and 1% penicillin–streptomycin. The SNU620 (ZQ1012), KATOIII (ZQ0238), and SNU5 (ZQ1044) cell lines were obtained from ZQXZBIO (Shanghai, China). SNU620 cells were cultured in RPMI-1640 medium, whereas KATOIII and SNU5 cells were maintained in IMDM (GUMD-B309; HyCyte^®^); all media were supplemented with 10% FBS and 1% penicillin–streptomycin. To generate luciferase-labeled cells for xenograft tumor establishment, SNU601 cells were transduced with pASLenti-pA-Luc2-CMV-EF1-EGFP-P2A-Puro-WPRE lentivirus through multiple rounds of infection, followed by puromycin selection (1 µg/mL) for 4 weeks to obtain stable SNU601-Luciferase cells.

### Source of primary human T cells

Primary human peripheral blood mononuclear cells (PBMCs) from healthy donors were obtained from Sailybio (China). Primary human T cells were then isolated from PBMCs using the EasySep™ Human T Cell Isolation Kit according to the manufacturer’s instructions (17951, STEMCELL).

### CAR design and vector construction

Two Claudin18.2-targeted CAR formats were generated. scFv-CAR: The Claudin18.2-binding scFv (designated Hu8E5) was derived from the Anti-Claudin18.2 antibody (Patent No.: US 11,089,118 B2). VHH-CAR: The Claudin18.2-specific humanized VHH (designated Hu7v3) was adapted from a published VHH-Fc format with confirmed specificity/affinity for Claudin18.2. The amino acid (or nucleotide) sequences of the scFv and VHH are provided in Supplementary Table (Supplementary Table 1).

For both CARs, the antigen-binding domain was fused in-frame to a CD28 hinge/transmembrane region, followed by costimulatory signaling elements (CD137/4-1BB and CD28) and a CD3ζ activation domain. Synthetic genes were produced by Sangon Biotech and subcloned into a pCDH lentiviral expression vector under a CMV promoter, yielding pCDH-Claudin18.2-scFv-CAR and pCDH-Claudin18.2-VHH-CAR (Supplementary Figure 1F). All plasmids were sequence-verified.

### Lentivirus packaging

HEK293T cells in a 15-cm culture dish were co-transfected with 18 µg of the lentiviral transfer vector, 12 µg of the packaging plasmid psPAX2 (Addgene, #12260), and 6 µg of the envelope plasmid pMD2.G (Addgene, #12259) using polyethyleneimine (PEI; Polysciences, #23966-1) as the transfection reagent. Culture supernatants were collected at 48 and 60 h post-transfection. Lentiviral particles were concentrated by ultracentrifugation (Beckman Coulter XPN-100) at 25,000 rpm for 2 h and stored at -80 °C until use.

### CAR-T cell generation

To generate human CAR-T cells, primary human T cells isolated from PBMCs were activated with Human T-Activator CD3/CD28 Dynabeads (11131D, Invitrogen) for 24 h and subsequently transduced with a CAR-encoding lentivirus. At 72 h post-transduction, CAR expression (transduction efficiency) in each CAR-T cell group was assessed by flow cytometry after staining with a fluorescent human full-length Claudin18.2 protein–VLP reagent (CL2-HF218; ACROBiosystems, USA).

Subsequently, to ensure matched expression levels for functional comparison, CAR-positive cells (Claudin18.2-VHH-CAR-T and Claudin18.2-scFv-CAR-T) were enriched by fluorescence-activated cell sorting or positive selection using the EasySep™ FITC Positive Selection Kit II (17682, STEMCELL Technologies), achieving > 90% purity as confirmed by flow cytometry. CAR-T cells were cultured in X-VIVO™ 15 medium (04-418Q, Lonza) supplemented with 10% FBS and recombinant human IL-2 (rhIL-2; 50 U/mL, 200-02-50; PeproTech) for subsequent experiments.

### Flow cytometry

All samples were acquired on a CytoFLEX or CytoFLEX LX flow cytometer (Beckman Coulter), and data were analyzed using FlowJo software (Tree Star). For surface staining, cells were incubated with the indicated antibodies in FACS buffer (PBS supplemented with 2% FBS) at 4 °C for 25 min. Cells were washed three times with FACS buffer and resuspended in PBS.

Antibodies used for staining included: CD3 PerCP (300428, BioLegend), CD45 PE-Cy7 (304016, BioLegend), CD4 APC (300514, BioLegend), CD8 FITC (301006, BioLegend), PD-1 APC (329908, BioLegend), PD-1 BV421 (329920, BioLegend), LAG-3 PE-Cy7 (369310, BioLegend), LAG-3 APC (369212, BioLegend), CD69 PE-Cy7 (310912, BioLegend), CD69 APC (310910, BioLegend), CD45RO APC (304210, BioLegend), CD62L PE (980710, BioLegend), Ki-67 FITC (151211/151212, BioLegend).

### CD69 activation readout (Jurkat assay)

According to previously published methods [29], Jurkat cells (1 × 10^5^) were co-incubated for 18 h with graded numbers of tumor cells (1 × 10^5^, 5 × 10^4^, 1 × 1^4^, 5 × 1^3^, 1 × 10^3^) in complete RPMI-1640 supplemented with IL-2 (30 ng/mL). After washing with PBS, cells were stained with anti-CD69 (BioLegend; #310906) for 30 min at 4 ℃, washed again, and analyzed by flow cytometry.

### In vitro cytotoxicity and cytokine secretion

Antitumor activity of non-transduced T cells (Control-T), Claudin18.2-VHH-CAR-T, and Claudin18.2-scFv-CAR-T cells was quantified in 24-h co-culture assays across effector-to-target (E: T) ratios ranging from 1:1 to 20:1. Target-cell lysis was determined using an LDH release assay (Promega). After 24 h of co-culture, supernatants were collected and lactate dehydrogenase (LDH) release was quantified using the CytoTox 96^®^ Non-Radioactive Cytotoxicity Assay (Promega). The percentage of cell death was calculated as follows: % cell death = [experimental LDH release (OD490) target-cell spontaneous release (OD490)]/[maximum LDH release (OD490) target-cell spontaneous release (OD490)]. Maximum LDH release was defined as the LDH measured from complete lysis of the target cells. Supernatants collected at 24 h were analyzed for IFN-γ and TNF-α by ELISA (eBioscience, China).

### Quantitative PCR

Total RNA was extracted from cells using the RNA simple Total RNA Kit (TIANGEN, Beijing, China). For each sample, 1 µg of RNA was reverse-transcribed into cDNA using the First-Strand cDNA Synthesis System (TIANGEN). Quantitative PCR was performed on a CFX96 Real-Time PCR System (Bio-Rad, USA) using SYBR Green PCR Master Mix (ABM, China), with 0.2 µg cDNA per reaction as the template. Target gene expression was normalized to GAPDH, and primer sequences are provided in (Supplementary Figure 1A).

### Western blotting

Cells were lysed in RIPA buffer (Beyotime, China; #P0013B) supplemented with protease inhibitors. Total protein was quantified using a BCA kit (Thermo Fisher; #23225). Equal amounts of protein (20–30 µg) were resolved on 10% or 12% SDS-PAGE gels (Epizyme, China; #PG112/PG113) with a prestained molecular weight ladder (Epizyme; #WJ103), transferred to PVDF membranes (Millipore; #IPVH00010), The membranes were then incubated with primary antibodies, including anti-Claudin18.2(HUABIO; ER1902-86), LAG-3/CD223 polyclonal antibody (Proteintech;16616-1-AP), TCF1/TCF7 polyclonal antibody (Proteintech;14464-1-AP), Granzyme M polyclonal antibody (Proteintech; 13208-1-AP), GAPDH monoclonal antibody (Proteintech; 60004-1-Ig). HRP-conjugated goat anti-rabbit IgG (Proteintech; SA00001-2), HRP-conjugated goat anti-mouse IgG, (Proteintech; SA00012-1) was used as the secondary antibody, followed by chemiluminescent detection.

### Xenograft model and in vivo treatment

Female NSG mice (NOD. Cg-Prkdc^scid^ Il2rg^tm1Wjl^/SzJ; 4–8 weeks; Vital River, Beijing, China) were maintained under SPF conditions at Yunnan University. Animals were randomized, and investigators were blinded to group allocation. Log-phase SNU601 cells were suspended in PBS containing 50% Matrigel at 2 × 10^7^ cells/mL, and 100 µL (2 × 10^6^ cells) was injected subcutaneously into the right axillary region. Mice (*n* = 5/group) received 1 × 10^7^ T cells (100 µL; Control-T, Claudin18.2-VHH-CAR-T, or Claudin18.2-scFv-CAR-T) via tail vein on days 16 and 30, following a two-dose schedule commonly used to maintain exposure during the expected post-infusion contraction phase. Tumor burden was monitored by weekly bioluminescence imaging. Mice were anesthetized with tribromoethanol (300 mg/kg, i.p.) and administered D-luciferin (150 mg/kg) prior to imaging on an IVIS Spectrum system (PerkinElmer; 1-min exposure; acquisition at ~ 3 min post-luciferin). Photon flux was quantified using Living Image software. Body weight was recorded weekly as a general safety metric. Humane endpoints were applied when control animals became moribund.

### In vivo trafficking and tumor infiltration analyses

At the study endpoint (Day 44), tumors were excised and weighed. Tumors were dissociated into single-cell suspensions using a Mouse Tumor Dissociation Kit (Miltenyi Biotec; #130-096-730), followed by Percoll density gradient enrichment. Tumor-infiltrating human T cells were quantified by flow cytometry using anti-human CD45-PE-Cy7, CD3-PerCP, CD8-FITC, and CD4-APC antibodies (as indicated), and CD4⁺/CD8⁺ CAR-T subsets were calculated accordingly. CAR copy number in tumor tissues was measured by RT-PCR at endpoint. For IHC, tumor samples were fixed in 4% paraformaldehyde and stained with anti-CD4 (EPR685; Abcam) and anti-CD8α (CAL66; Abcam; Servicebio, Wuhan, China). Positive areas were quantified using ImageJ.

### Histopathology

Heart and liver were collected on Day 44, fixed in 10% neutral buffered formalin for 48 h, embedded in paraffin, sectioned at 5 μm, and stained with hematoxylin and eosin using routine procedures.

### Coculture for proliferation and exhaustion marker kinetics

Tumor cells were plated in 24-well plates and allowed to adhere for 24 h. CAR-T cells were then added at the specified E: T ratios in complete medium containing 10% FBS and recombinant human IL-2 (50 IU/mL) and co-cultured for 48 h. CAR-T cells were harvested to quantify expansion and to longitudinally assess exhaustion-associated markers (PD-1 and LAG-3). Fold expansion was calculated as the cumulative CAR-T cell number normalized to the number initially seeded (day 0/first coculture).

### CAR-T cell enumeration

In vitro: CAR-T cell numbers were calculated as (CAR-positive fraction) × (total viable cell count). Viability was determined by trypan blue exclusion using a Countstar^®^ automated cell counter (ALIT Life Science), and CAR positivity was measured by flow cytometry. In vivo: Flow-sorted CAR-T cells (purity > 90%) were infused. At the indicated time points, tumors were mechanically/enzymatically dissociated and analyzed by flow cytometry; the total number of human CD3⁺ events were used as a surrogate for intratumoral CAR-T abundance.

### RNA sequencing

VHH-CAR-T and scFv-CAR-T cells were stimulated with antigen in vitro for 48 h prior to RNA-seq. Total RNA was extracted using TRIzol (Takara), snap-frozen in RNase-free tubes in liquid nitrogen, and quality-controlled on an Agilent 2100 Bioanalyzer using the RNA Nano 6000 kit. Libraries passing QC were quantified (Qubit 2.0) and sequenced on an Illumina NovaSeq 6000 platform (150-bp paired-end; Novogene).

#### Bulk RNA-seq processing and downstream analyses

Adapter and quality trimming were performed with Trimmomatic (v0.38) [47]. Clean reads were mapped to GRCh38 using HISAT2 (v2.1.0). Gene-level read counts were generated with HTSeq (v0.11.2) using “mode intersection-strict stranded no minaqual”. Differential expression was assessed with edgeR (v3.22.3) using thresholds of fold change > 2 and FDR < 0.05 [48]. PCA was conducted using FactoMineR (v2.4). KEGG enrichment was performed with clusterProfiler (v3.12). GSEA was used to test coordinated pathway-level differences, and gene sets with FDR < 0.05 were considered significant.

### Structural modeling and molecular dynamics simulations

Structural models of Claudin18.2 in complex with either the VHH or scFv recognition domain were generated using AlphaFold 3. The top-ranked models were selected based on prediction confidence and inspection of the antigen-binding interface. Protein–protein interfaces were analyzed using PDBePISA to calculate interface area, ΔiG_total, buried solvent-accessible surface area, interface residues, and hydrogen bonds. Molecular graphics were prepared using PyMOL.

Molecular dynamics simulations were performed using GROMACS. Each complex was solvated in a truncated octahedral box with TIP3P water molecules, neutralized with Na⁺/Cl⁻ ions, and parameterized using the AMBER99SB-ILDN force field. After energy minimization and NVT/NPT equilibration, 200-ns production simulations were conducted with a 2-fs time step. Long-range electrostatic interactions were calculated using the particle mesh Ewald method, with a 1.0-nm cutoff for van der Waals and short-range Coulomb interactions. RMSD and B-factor-like displacement analyses were performed to evaluate complex stability and residue-level flexibility.

### Statistical analysis

Flow cytometry data were analyzed in FlowJo (v10). Western blot band intensity was quantified using ImageJ. Statistical analyses were performed in GraphPad Prism (v9). Two-group comparisons were evaluated using unpaired two-tailed Student’s t-tests. Multi-group comparisons were analyzed using one-way or two-way ANOVA followed by Tukey’s post hoc test for multiple comparison corrections, as specified in the respective figure legends. Survival curves were analyzed using the log-rank (Mantel-Cox) test. Data are presented as mean ± SEM. **Exact P-values are reported where applicable, and a P-value < 0.05 was considered statistically significant (*P < 0.05, **P < 0.01, ***P < 0.001, ****P < 0.0001; ns, not significant).

## Supplementary Information

Below is the link to the electronic supplementary material.

Supplementary Material 1

## Data Availability

All data generated or analyzed during this study are included in this article and additional data are available from the corresponding author upon reasonable request.
