# Supplementary material for: VHH-based antigen recognition programs Claudin18.2 CAR-T cells for durable activity in low-antigen solid tumors
Source: J Transl Med. 2026 Jul 22;24:1319. doi: 10.1186/s12967-026-08611-2 (PMC13681878; doi:10.1186/s12967-026-08611-2)
Supplement: Supplementary file 1 [file 12967_2026_8611_MOESM1_ESM.docx]

**VHH-based antigen recognition programs Claudin18.2 CAR-T cells for durable activity in low-antigen solid tumors**

Dan He^1#^, Xiaoyuan Hu ^1#^, Jiyin Guo^1^, Jiaxing Cheng^1^, Weike Li^1^, Sidi Wan^1^, Yunshan Ye^1^, Lei Zhu^1^, Li Li^1^, Ke Li^1^, Chunlei Ge^1^, Tao Li^1*^, Hong Yao^1*^.

^1^Cancer Biotherapy Center & Cancer Research Institute, The Third Affiliated Hospital of Kunming Medical University, Yunnan Cancer Hospital, Peking University Cancer Hospital Yunnan, Kunming, China.

***Corresponding Author:** Hong Yao, E-mail address: [yaohong20055@hotmail.com](mailto:yaohong20055@hotmail.com);

Tao Li, E-mail address: [litaove@163.com](mailto:litaove@163.com);

\# Dan He and Xiaoyuan Hu contributed equally to this manuscript.

**Supplementary Figure**

Figure S1. Construction of Claudin18.2-targeting CAR lentiviral vectors and optimization of Jurkat transduction conditions. (A) qRT–PCR primer sequences used for quantifying CLDN18.2 mRNA: Forward, TTGGGATTGCGGGCATCATT; Reverse, CAGCTGTTACGGGGTTGTTG. (B) SnapGene maps of the lentiviral transfer plasmids encoding Claudin18.2-VHH-CAR (left) and Claudin18.2-scFv-CAR (right), both based on the pCDH backbone and carrying a puromycin resistance cassette (Puro). Plasmid sizes are ~7893 bp (VHH-CAR) and ~7593 bp (scFv-CAR). Key vector elements and CAR modules are annotated on the maps. (C) Flow cytometric assessment of CAR surface expression in Jurkat cells transduced at different multiplicities of infection (MOI = 0, 10, 20, 40, 80). Representative FITC histograms are shown with the positivity threshold indicated by the dashed line; the corresponding CAR⁺ percentages are labeled. CAR⁺ frequencies for scFv-CAR-Jurkat were 1.83%, 48.1%, 59.7%, 59.2%, and 61.0%, and for VHH-CAR-Jurkat were 1.83%, 51.5%, 51.5%, 55.5%, and 54.1% at MOI 0, 10, 20, 40, and 80, respectively. (D) Representative gating strategy corresponding to (C). Lymphocytes were first gated on FSC-A vs SSC-A, followed by identification of FITC-positive events (CAR-expressing cells) on FITC-A vs SSC-A, with FITC-A histograms illustrating the distribution and frequency of CAR⁺ cells across MOI conditions.

Figure S2. Representative flow-cytometry gating strategy for assessing early activation (CD69) in Claudin18.2-targeting CAR-Jurkat cells. Representative gating is shown for VHH-CAR-Jurkat stained for CD69 (APC channel) using different input cell numbers (1×10^3^, 1×10^4^, and 5×10^4^). Left panels: lymphocytes were gated on FSC-A vs SSC-A. Middle panels: CD69⁺ events were defined on APC-A vs SSC-A (APC-A subset gate). Right panels: APC-A histograms display the CD69 fluorescence distribution with the CD69⁺ gate/threshold and the corresponding CD69⁺ percentages annotated. This gating scheme was applied throughout subsequent CAR-Jurkat co-culture assays to ensure consistent quantification of antigen-induced early activation across CAR formats.

Figure S3. Representative flow-cytometry gating strategy for assessing early activation (CD69) in Claudin18.2-targeting CAR-Jurkat cells. Representative gating is shown for scFv-CAR-Jurkat stained for CD69 (APC channel) using different input cell numbers (1×10^3^, 1×10^4^, and 5×10^4^). Left panels: lymphocytes were gated on FSC-A vs SSC-A. Middle panels: CD69⁺ events were defined on APC-A vs SSC-A (APC-A subset gate). Right panels: APC-A histograms display the CD69 fluorescence distribution with the CD69⁺ gate/threshold and the corresponding CD69⁺ percentages annotated. This gating scheme was applied throughout subsequent CAR-Jurkat co-culture assays to ensure consistent quantification of antigen-induced early activation across CAR formats.

Figure S4. No overt systemic toxicity was observed for Claudin18.2-targeting CAR-T treatment in the NSG xenograft model.

(A) At the study endpoint (Day 44) of the luc-SNU601 subcutaneous xenograft experiment, mice receiving Control-T, scFv-CAR-T, or VHH-CAR-T were euthanized and major organs (heart, liver, spleen, lungs, and kidney) were collected for H&E staining. Representative histology images are shown, with no apparent inflammatory infiltration, necrosis, or gross tissue damage; scale bar, 100 μm.

(B) Body weight was monitored longitudinally during treatments. Body weights remained stable across groups with no significant differences (ns, as indicated). Data are presented as mean ± SEM.

Figure S5. Enhanced proliferation profile and serial cytotoxicity of VHH-CAR-T cells under antigen stimulation.

(A) Transcriptional expression levels of the cell-cycle marker *MKI67* in Claudin18.2-scFv-CAR-T and Claudin18.2-VHH-CAR-T cells determined by RNA-seq after a 48-hour antigen stimulation, expressed as Log2(FPKM+1) values (n = 3 biological replicates per group).

(B) Representative flow cytometry histogram (left) and statistical quantification (right) of intracellular Ki-67 expression in the indicated CAR-T cells after a 48-hour co-culture with SNU601 gastric cancer cells at an effector-to-target (E: T) ratio of 1:5 (n = 3).

(C) In vitro cumulative cytotoxicity of Control-T, scFv-CAR-T, and VHH-CAR-T cells evaluated by standard lysis assay against SNU601 cells across three consecutive rounds of antigen challenges (designated as sti-1, sti-2, and sti-3). For each stimulation round, CAR-T cells were challenged at an E:T ratio of 20:1 for 24 h.

Data are presented as mean $\pm$ SEM. Statistical significance was assessed using an unpaired two-tailed Student’s t-test (A, B) or two-way ANOVA followed by Tukey's post-hoc test (C). ns, not significant; **P* < 0.05, *P* < 0.01, *****P* < 0.0001.

**Supplementary Table1 Sequences of Claudin18.2 scFv CAR and VHH CAR**

| CAR | Gene Sequence |
| --- | --- |
| Cluadin18.2  scFv CAR | TCTAGAGCCACCATGGCCTTACCAGTGACCGCCTTGCTCCTGCCGCTGGCCTTGCTGCTCCACGCCGCCAGGCCGGACATCGTGATGACACAATCTCCAGACAGCCTCGCAGTTTCCCTTGGGGAGCGGGCCACGATTAACTGTAAGTCCAGTCAGAGCCTGTTCAATAGCGGTAACCAGAAAAACTACCTGACATGGTATCAACAGAAACCAGGTCAGCCACCCAAACTCCTGATCTATTGGGCTTCTACAAGGGAAAGTGGCGTGCCTGATAGATTTAGTGGAAGTGGAAGTGGCACCGACTTTACGCTTACCATTAGTTCCCTTCAAGCAGAAGATGTGGCTGTTTACTATTGCCAAAACGCCTACAGCTTTCCGTATACTTTCGGTGGCGGTACCAAGCTGGAGATCAAGGGCGGTGGCGGTTCTGGTGGCGGTGGCTCCGGCGGTGGCGGTTCTCAGGTGCAATTGCAAGAAAGCGGACCCGGCCTGATTAAACCTTCCCAGACTCTTAGCCTGACTTGCACAGTAAGCGGGGGCTCCATTTCTTCTGGCTATAACTGGCACTGGATCAGGCAACCTCCTGGAAAAGGGCTTGAGTGGATCGGCTATATCCACTATACCGGCTCCACTAACTATAATCCCGCCTTGCGGTCACGCGTCACAATTTCCGTGGATACCTCCAAGAATCAGTTTAGCCTTAAACTCTCTTCAGTCACAGCTGCGGACACTGCCATTTATTATTGCGCCCGCATCTACAACGGGAACAGCTTTCCATATTGGGGACAGGGTACCACAGTAACAGTTGCGGCCGC. |
| Cluadin18.2 VHH CAR | GACAAGACCCACACCTGTCCTCCATGTCCTGCTCCAGAACTGCTCGGCGGACCTTCCGTGTTTCTGTTCCCTCCAAAGCCTAAGGACACCCTGATGATCAGCAGAACCCCTGAAGTGACCTGCGTGGTGGTGGATGTGTCCCACGAGGACCCCGAAGTGAAGTTCAATTGGTACGTGGACGGCGTGGAAGTGCACAACGCCAAGACCAAGCCTAGAGAGGAACAGTACAACAGCACCTACAGAGTGGTGTCCGTGCTGACAGTGCTGCATCAGGACTGGCTGAACGGCAAAGAGTACAAGTGCAAGGTGTCCAACAAGGCCCTGCCTGCTCCTATCGAGAAAACCATCAGCAAGGCCAAGGGCCAGCCTAGGGAACCCCAGGTTTACACACTGCCTCCAAGCAGGGACGAGCTGACCAAGAATCAGGTGTCCCTGACCTGCCTCGTGAAGGGCTTCTACCCTTCCGATATCGCCGTGGAATGGGAGAGCAATGGCCAGCCTGAGAACAACTACAAGACAACCCCTCCTGTGCTGGACAGCGACGGCTCATTCTTCCTGTACAGCAAGCTGACCGTGGACAAGTCCAGATGGCAGCAGGGCAACGTGTTCAGCTGCTCCGTGATGCACGAGGCCCTGCACAACCACTACACCCAGAAGTCCCTGAGCCTGTCTCCTGGCAAA. |
